# Regulation of reactive oxygen species-mediated abscisic acid signaling in guard cells and drought tolerance by glutathione

**DOI:** 10.3389/fpls.2013.00472

**Published:** 2013-11-20

**Authors:** Shintaro Munemasa, Daichi Muroyama, Hiroki Nagahashi, Yoshimasa Nakamura, Izumi C. Mori, Yoshiyuki Murata

**Affiliations:** ^1^Division of Agricultural and Life Science, Graduate School of Environmental and Life Science, Okayama UniversityOkayama, Japan; ^2^Faculty of Agriculture, Okayama UniversityOkayama, Japan; ^3^Institute of Plant Science and Resources, Okayama UniversityKurashiki, Japan

**Keywords:** abscisic acid, glutathione, reactive oxygen species, guard cell, stomata

## Abstract

The phytohormone abscisic acid (ABA) induces stomatal closure in response to drought stress, leading to reduction of transpirational water loss. A thiol tripeptide glutathione (GSH) is an important regulator of cellular redox homeostasis in plants. Although it has been shown that cellular redox state of guard cells controls ABA-mediated stomatal closure, roles of GSH in guard cell ABA signaling were largely unknown. Recently we demonstrated that GSH functions as a negative regulator of ABA signaling in guard cells. In this study we performed more detailed analyses to reveal how GSH regulates guard cell ABA signaling using the GSH-deficient Arabidopsis mutant *cad2-1*. The *cad2-1* mutant exhibited reduced water loss from rosette leaves. Whole-cell current recording using patch clamp technique revealed that the *cad2-1* mutation did not affect ABA regulation of S-type anion channels. We found enhanced activation of Ca^2+^ permeable channels by hydrogen peroxide (H_2_O_2_) in *cad2-1* guard cells. The *cad2-1* mutant showed enhanced H_2_O_2_-induced stomatal closure and significant increase of ROS accumulation in whole leaves in response to ABA. Our findings provide a new understanding of guard cell ABA signaling and a new strategy to improve plant drought tolerance.

## Introduction

A phytohormone abscisic acid (ABA) induces closing of stomatal pores on leaf epidermis, resulting in reduction of transpirational water loss. The central ABA signaling module is composed of ABA receptors PYR/PYL/RCAR, clade A type 2C protein phosphatases (PP2Cs), and subclass 2 of Snf1-related kinases (SnRK2s) and regulates downstream targets (Fujii et al., 2009; Ma et al., 2009; Park et al., 2009) including ion channels (Geiger et al., 2009; Lee et al., 2009).

Activation of slow type (S-type) anion channels is a key step for ABA signaling in guard cells and drives depolarization of plasma membrane of guard cells, which in turn evokes K^+^ extrusion (Linder and Raschke, 1992; Schroeder and Keller, 1992). A guard cell plasma membrane protein SLAC1 represents the S-type anion channel activity (Negi et al., 2008; Vahisalu et al., 2008). It has been demonstrated that ABA activation of S-type anion channels is mediated via a cytosolic Ca^2+^-dependent pathway (Marten et al., 2007; Siegel et al., 2009). ABA activates hyperpolarization-activated Ca^2+^-permeable cation (I_Ca_) channels in the plasma membrane of guard cells (Schroeder and Hagiwara, 1990; Hamilton et al., 2000; Pei et al., 2000; Kwak et al., 2003) and induces elevation of cytosolic free Ca^2+^ concentration ([Ca^2+^]_cyt_) in guard cells (McAinsh et al., 1990; Schroeder and Hagiwara, 1990; Gilroy et al., 1991; Allan et al., 1994; Grabov and Blatt, 1998; Allen et al., 1999; Marten et al., 2007). The [Ca^2+^]_cyt_ signals are decoded by Ca^2+^ sensor proteins such as calcium dependent protein kinases (CDPKs). Electrophysiology experiments using *Xenopus* oocyte demonstrated that Arabidopsis CDPKs, CPK6, CPK21, and CPK23, directly phosphorylate and activate SLAC1 channel (Geiger et al., 2010; Brandt et al., 2012).

It has been suggested that guard cell ABA signaling involves redox regulation. Reactive oxygen species (ROS) including hydrogen peroxide (H_2_O_2_) serve as a key mediator of ABA activation of I_Ca_ channels (Pei et al., 2000; Murata et al., 2001; Kwak et al., 2003). Exogenous application of H_2_O_2_ activates I_Ca_ channels and evokes guard cell [Ca^2+^]_cyt_ increases (Pei et al., 2000). Plasma membrane NAD(P)H oxidases are responsible for ABA-induced ROS production in guard cells (Kwak et al., 2003). Arabidopsis glutathione peroxidase 3 (AtGPX3) was shown to function as both a ROS scavenger and a ROS signal transducer in ABA signaling (Miao et al., 2006). Emerging evidences suggest that ROS production by apoplastic enzymes such as cell-wall bound peroxidases is also involved in induction of stomatal closure (An et al., 2008; Khokon et al., 2011; Hossain et al., 2013).

Glutathione (GSH) is the most abundant non-protein thiol compound in plants and a key regulator of cellular redox homeostasis. GSH is involved in various physiological processes including growth, development, and defense response to biotic and abiotic stresses (May et al., 1998; Noctor and Foyer, 1998). Previously we reported that ABA as well as methyl jasmonate (MeJA) decreases the GSH contents of guard cells (Akter et al., 2010; Okuma et al., 2011). Arabidopsis GSH-deficient mutants, *chl-1* and *cad2-1* exhibit enhanced ABA-induced and MeJA-induced stomatal closure and a membrane permeable derivative of GSH, GSH monoethyl ester (GSHmee) restored the stomatal phenotype of *chl-1* and *cad2-1* mutants (An et al., 2008; Akter et al., 2010, 2012, 2013; Okuma et al., 2011), demonstrating that GSH functions as a negative regulator of ABA and MeJA signaling in guard cells. However, the detailed mechanism of how GSH modulates the guard cell responses is still unclear.

In this study, we analyzed GSH regulation of ABA signaling in guard cells using the Arabidopsis GSH-deficient mutant *cad2-1*. The *cad2-1* mutant is deficient in the first GSH biosynthesis enzyme, γ-glutamylcysteine synthetase. We found that the *cad2-1* mutation causes enhanced ROS activation of I_Ca_ channel and ABA-induced ROS accumulation in apoplast. A new signal model for regulation of ROS-mediated ABA signaling by GSH in guard cells is also proposed.

## Materials and methods

### Plant material and growth

The Arabidopsis ecotype Columbia (Col) and *cad2-1* mutant plants were grown on soil mixture of 70% (v/v) vermiculite (Asahi-kogyo, Okayama, Japan) and 30% (v/v) Sakata Supermix-A (Sakata Seed Corporation, Yokohama, Japan) in growth chambers at 21°C under a 16-h-light/8-h-dark photoperiod with photon flux density of 80 μmol m^−2^ s^−1^. Four- to six-week-old plants were used in all experiments.

### Water loss assay

Three detached rosette leaves were placed on a plastic tray and the loss in fresh weight was monitored at the indicated times.

### Stomatal aperture measurements

Stomatal aperture measurements were performed as described previously (Munemasa et al., 2007; Okuma et al., 2011). Detached rosette leaves were floated on stomatal assay buffer containing 5 mM KCl, 50 μM CaCl_2_, and 10 mM MES-Tris (pH 5.6) for 2 h in the light to induce stomatal opening, followed by the addition of H_2_O_2_. After 2-h incubation in the light, the leaves were shredded and epidermal tissues were collected. At least 20 stomatal apertures were measured on each individual experiment.

### Electrophysiology

Guard cell protoplasts (GCPs) were prepared from Arabidopsis rosette leaves by the enzymatic method, as described previously (Pei et al., 1997). Whole-cell currents were recorded using patch clamp technique, as described previously (Munemasa et al., 2007, 2011). For S-type anion current measurements, the pipette solution contained 150 mM CsCl, 2 mM MgCl_2_, 6.7 mM EGTA, 5.58 mM CaCl_2_ (free Ca^2+^ concentration: 2 μM), and 10 mM HEPES-Tris (pH 7.1). The bath solution contained 30 mM CsCl, 2 mM MgCl_2_, 1 mM CaCl_2_, and 10 mM MES-Tris (pH 5.6). 5 mM Mg-ATP was freshly added to the pipette solution before experiments. For I_Ca_ current measurements, the pipette solution contained 10 mM BaCl_2_, 4 mM EGTA, and 10 mM HEPES-Tris (pH 7.1). The bath solution contained 100 mM BaCl_2_, and 10 mM MES-Tris (pH 5.6). 0.1 mM DTT was added to both pipette and bath solutions freshly before experiments. In both cases, osmolarity was adjusted to 500 mmol/kg (pipette solutions) and 485 mmol/kg (bath solutions) with D-sorbitol.

### Detection of ROS accumulation in whole leaves

Accumulation of H_2_O_2_ in whole leaves was monitored using 3,3′-diaminobenzidine (DAB) according to Maruta et al. (2010) with slight modifications. Detached rosette leaves were vacuum infiltrated with DAB assay buffer containing 1 mg mL^−1^ DAB, 5 mM KCl, 50 μM CaCl_2_, and 10 mM MES-Tris (pH 5.6). Infiltrated leaves are incubated in DAB assay buffer with or without ABA for 4 h in the light. The leaves were then decolorized by boiling in ethanol. Apoplastic ROS were visualized as a reddish-brown color and quantified using Image-J software (National Institutes of Health, USA).

### Statistical analysis

All statistical significance was analyzed by double-tailed Student's *t*-test. We regarded differences at the level of *P* < 0.05 as significant.

## Results

### The *cad2-1* mutant showed enhanced drought tolerance

**Figure 1:**
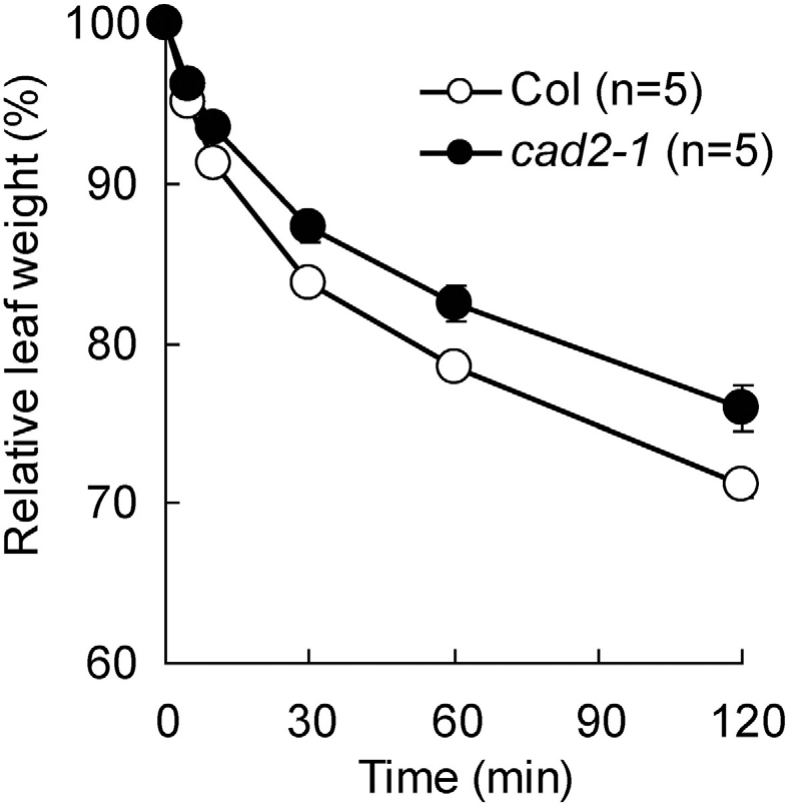
**The *cad2-1* mutant shows reduced transpirational water loss.** Fresh weight loss of detached rosette leaves. The bars represent the mean ± *SE* values of five independent replicates.

Previously we reported that GSH depletion by the *cad2-1* mutation enhances ABA-induced stomatal closure in Arabidopsis (Okuma et al., 2011). To assess effect of the *cad2-1* mutation on drought tolerance, we monitored water loss from detached rosette leaves. As shown in Figure 1, compared to wild type, the *cad2-1* mutant exhibited reduced water loss from detached rosette leaves (*P* < 0.018 for Col *vs*. *cad2-1* at 120 min after leaf detachment). This result suggests that the *cad2-1* mutation improves drought tolerance.

### Effect of the *cad2-1* mutation on ABA regulation of s-type anion channels.

**Figure 2:**
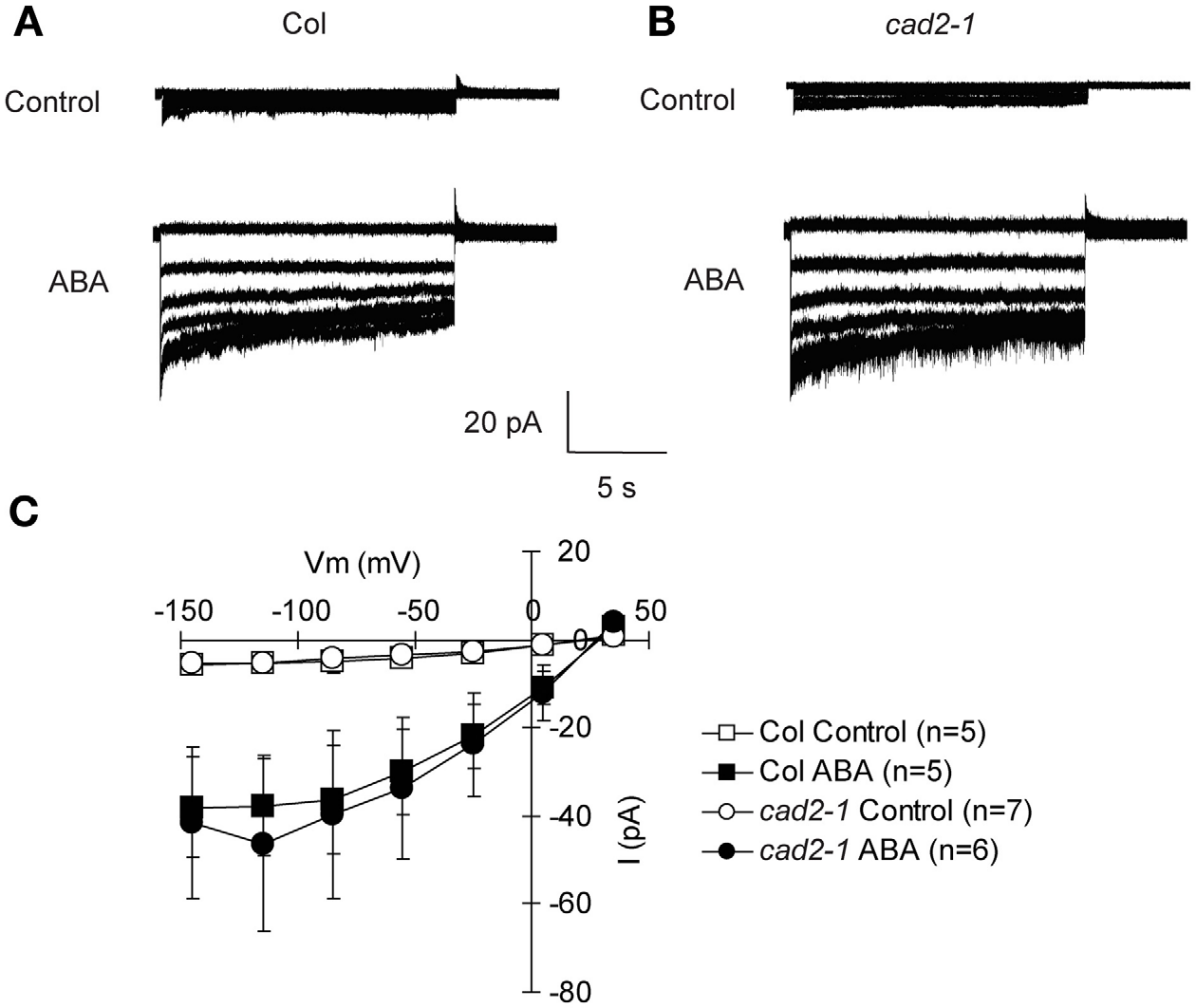
**Identical ABA activation of S-type anion channel currents in wild-type GCPs and *cad2-1* GCPs. (A)** Representative current traces of wild-type GCPs without ABA (upper trace) or with 10 μM ABA (Lower trace). **(B)** Representative current traces of *cad2-1* GCPs without ABA (upper trace) or with 10 μM ABA (Lower trace). **(C)** Average current-voltage curves of wild-type GCPs and *cad2-1* GCPs as recorded in **(A)** and **(B)**. The voltage protocol was stepped-up from +35 to −145 mV in 30-mV decrements (holding potential: +30 mV). GCPs were treated with 10 μM ABA for 30 min before recordings. The bars represent the mean ± *SE* values of at least five independent replicates.

The *cad2-1* mutation does not affect ABA activation of I_Ca_ channels, suggesting that GSH functions downstream of [Ca^2+^]_cyt_ elevation in guard cell ABA signaling (Okuma et al., 2011). Activation of S-type anion channel is mediated by [Ca^2+^]_cyt_ elevation and considered as a crucial step for ABA-, MeJA-, and CO_2_-induced stomatal closure (Schroeder and Hagiwara, 1989; Pei et al., 1997; Munemasa et al., 2007; Hu et al., 2010). To confirm whether GSH depletion in the *cad2-1* mutant affects ABA regulation of S-type anion channel activity, whole-cell patch-clamp analysis was performed. S-type anion currents were observed in wild-type GCPs pretreated with 10 μM ABA (*P* < 0.023 for Col Control *vs*. Col ABA at −135 mV; Figure 2). We also found that ABA evoked S-type anion currents in *cad2-1* GCPs to the same extent as in wild-type GCPs (*P* < 0.045 for *cad2-1* Control *vs*. *cad2-1* ABA at −135 mV; Figure 2). Note that in our experimental condition, free Ca^2+^ concentration in the pipette solution was buffered to 2 μM and no obvious S-type anion currents were observed in both wild-type guard cells and *cad2-1* guard cells without ABA pretreatment.

### The *cad2-1* mutation enhanced H_2_O_2_ activation of I_Ca_ channels

**Figure 3:**
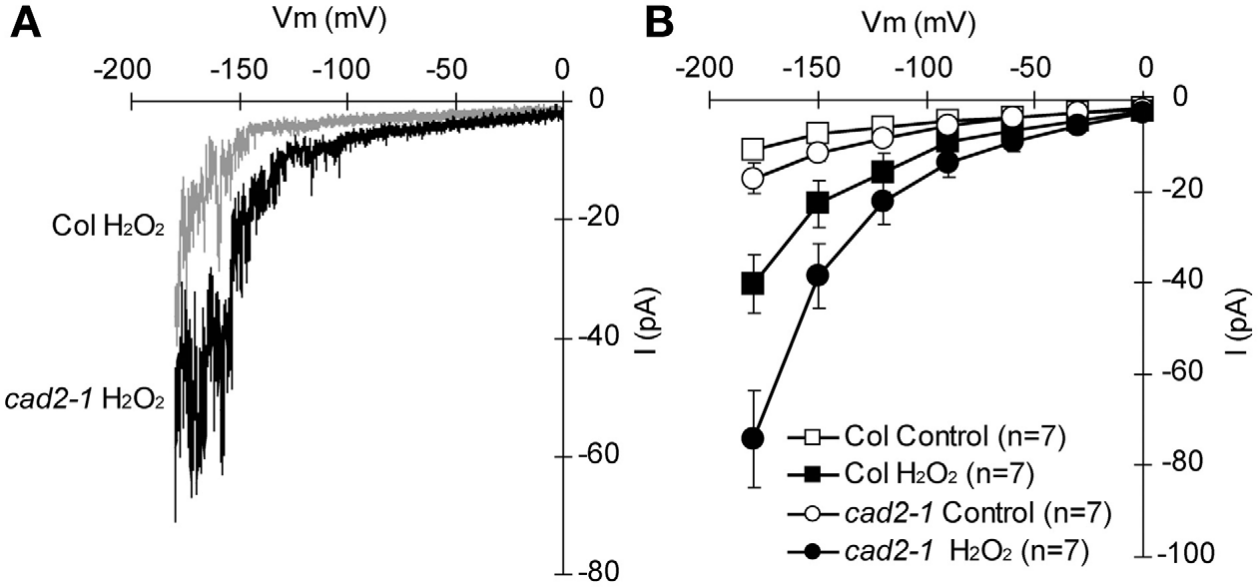
**Enhanced activation of *cad2-1* I_Ca_ channel currents by H_2_O_2_. (A)** Representative current traces of 1 mM H_2_O_2_-activated I_Ca_ currents of wild-type GCPs (gray) and *cad2-1* GCPs (black). **(B)** Average current-voltage curves of wild-type GCPs and *cad2-1* GCPs as recorded in **(A)**. A voltage ramp protocol from 0 to −180 mV was used (holding potential, 0 mV; ramp speed, 200 mV sec^−1^). GCPs were treated with 1 mM H_2_O_2_ for 3 min before recordings. The bars represent the mean ± *SE* values of seven independent replicates.

**Figure 4:**
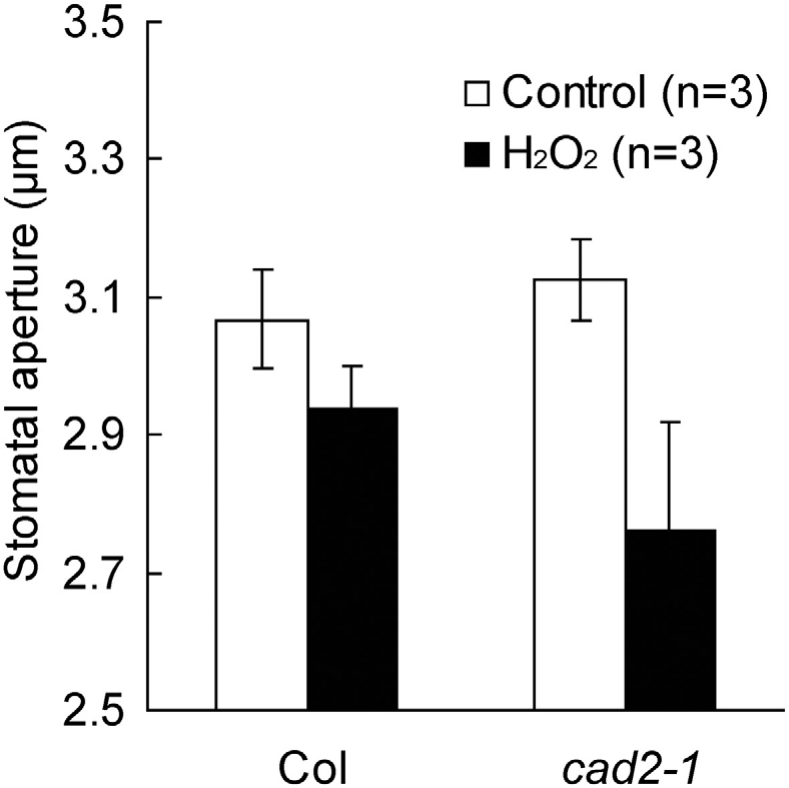
**Stomata of the *cad2-1* mutant are more sensitive to exogenous H_2_O_2_ than those of wild type.** Stomatal apertures of wild type and the *cad2-1* mutant were measured 2 h after 100 μM H_2_O_2_ application. Twenty averages from three independent experiments (60 total stomata per bar) are shown. The bars represent the mean ± *SE* values.

ABA activation of I_Ca_ channels involves ROS as a second messenger (Pei et al., 2000; Murata et al., 2001; Kwak et al., 2003). Depletion of the major redox regulator, GSH, in the *cad2-1* mutant might affect ROS-mediated ABA signaling in guard cells. Previously we demonstrated that ABA activation of I_Ca_ channels is not enhanced in *cad2-1* guard cells (Okuma et al., 2011). However, ROS activation of I_Ca_ channels in *cad2-1* guard cells was not yet analyzed. We examined effects of the *cad2-1* mutation on ROS regulation of I_Ca_ channels in guard cells. H_2_O_2_ at 1 mM activates hyperpolarization-activated currents in both wild-type GCPs and *cad2-1* GCPs (Figure 3). The H_2_O_2_ activation of I_Ca_ currents was enhanced in *cad2-1* GCPs compared to wild-type GCPs (*P* < 0.018 for Col H_2_O_2_ *vs*. *cad2-1* H_2_O_2_ at −180 mV; Figure 3). To examine effects of the enhanced H_2_O_2_ activation of *cad2-1* I_Ca_ channels on H_2_O_2_-induced stomatal closure, we performed stomatal bioassay. Significant reduction of stomatal apertures was observed in 100 μM H_2_O_2_-treated *cad2-1* mutant (*P* < 0.038 for *cad2-1* Control *vs*. *cad2-1* H_2_O_2_; Figure 4) but not wild type (*P* = 0.13 for Col Control *vs*. Col H_2_O_2_; Figure 4). Moreover, we found that H_2_O_2_-induced stomatal closure in the *cad2-1* mutant was significantly attenuated by a membrane permeable derivative of GSH, GSHmee (*P* < 0.047 for H_2_O_2_ without GSHmee *vs*. H_2_O_2_ with GSHmee; Figure S1). These results suggest that decreased GSH contents in *cad2-1* guard cells confer enhanced stomatal response to H_2_O_2_.

### The *cad2-1* mutant showed ABA-induced apoplastic ROS accumulation in leaves

**Figure 5:**
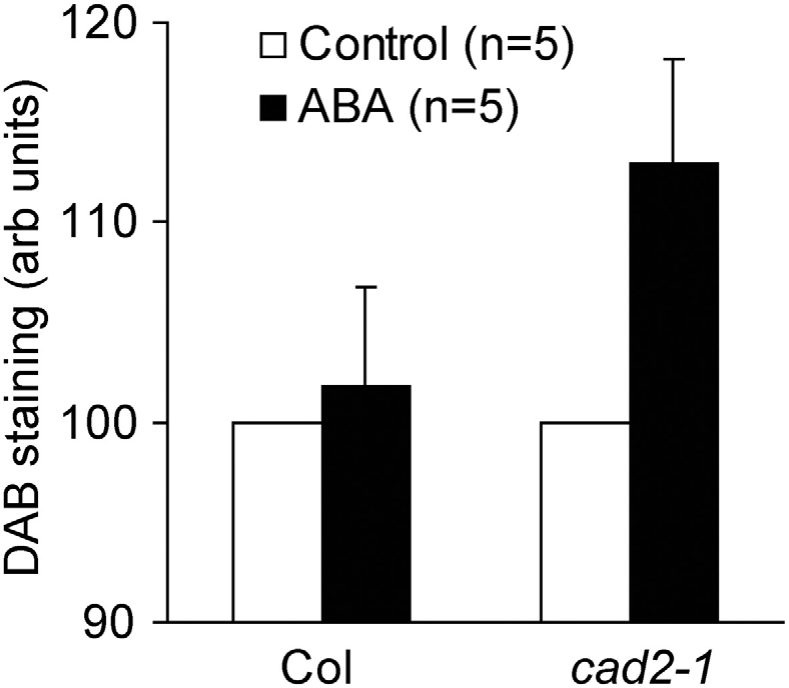
**ABA induces ROS accumulation in whole leaves of the *cad2-1* mutant but not in whole leaves of wild type.** Accumulation of ROS in whole leaves was monitored using DAB. The vertical scale represents the relative values of pixel intensity of DAB brown color when the values of 50 μM ABA treated leaves are normalized to control value taken as 100 for each experiment. Each datum was obtained from at least four leaves. The bars represent the mean ± *SE* values of five independent replicates.

Accumulation of ROS in guard cell cytosol occurs during ABA-induced stomatal closure (Pei et al., 2000; Kwak et al., 2003). Previously we revealed that ABA-induced ROS accumulation in guard cell cytosol was not altered by GSH depletion (Jahan et al., 2008; Okuma et al., 2011; Akter et al., 2012). In addition to ROS produced by plasma membrane NAD(P)H oxidases, ROS produced by apoplastic enzymes such as cell-wall bound peroxidases participate in regulation of stomatal movement (An et al., 2008; Khokon et al., 2011; Hossain et al., 2013). To examine whether apoplastic ROS accumulation contributes to guard cell ABA signaling, we performed DAB staining experiments, which allows us to monitor ROS produced by apoplastic enzymes as well as ROS produced by NAD(P)H oxidases (Bindschedler et al., 2006; Khokon et al., 2011; Hossain et al., 2013). Wild-type leaves did not exhibit apoplastic ROS accumulation even when treated with high concentration of ABA (50 μM) (*P* = 0.72 for Col Control *vs*. Col ABA; Figure 5). However, apoplastic ROS accumulation was significantly increased by ABA in *cad2-1* leaves (*P* < 0.04 for *cad2-1* Control *vs*. *cad2-1* ABA; Figure 5). This result suggests that the accumulation of apoplastic ROS contributes to enhanced ABA response of *cad2-1* guard cells.

## Discussion

Depletion of GSH enhances ABA-induced stomatal closure (Jahan et al., 2008; Okuma et al., 2011; Akter et al., 2012). However, the mechanism of how GSH regulates guard cell ABA signaling and involvement of GSH in controlling transpirational water loss have been unclear. In this manuscript, we tested involvement of GSH in controlling water loss from leaves and performed the detailed analysis of GSH regulation of guard cell ABA signaling using the GSH-deficient *cad2-1* mutant. We confirmed that the *cad2-1* mutation caused not only enhanced ABA-induced stomatal closure (Okuma et al., 2011) but also reduction of water loss from leaves (Figure 1). Hence manipulation of GSH level might provide a new strategy to improve plant drought tolerance.

ABA activation of S-type anion channels is not altered in the *cad2-1* guard cells (Figure 2). It has been suggested that the elevated [Ca^2+^]_cyt_ is required for ABA activation of S-type anion channels and ABA “primes” Ca^2+^ sensitivity of S-type anion channels (Siegel et al., 2009). In this study, we used the pipette solution where free [Ca^2+^] was buffered to 2 μM (See Materials and Methods). No obvious S-type anion current was observed in *cad2-1* guard cells as well as wild-type guard cells without ABA pretreatment (Figure 2), suggesting that the *cad2-1* mutation does not affect the priming state of Ca^2+^ sensitivity of S-type anion channels.

ABA induces guard cell [Ca^2+^]_cyt_ elevation via activation of plasma membrane I_Ca_ channels (Hamilton et al., 2000; Pei et al., 2000). Previously we found identical ABA activation of I_Ca_ channels in wild-type and *cad2-1* guard cells (Okuma et al., 2011). ROS mediates ABA activation of I_Ca_ channels (Pei et al., 2000; Murata et al., 2001; Kwak et al., 2003). In this study, we found enhanced response of *cad2-1* I_Ca_ channels to exogenous H_2_O_2_ (Figure 3). It was reported that ozone, an elicitor of ROS, induces biphasic [Ca^2+^]_cyt_ elevation in seedlings and depletion of GSH by the *cad2-1* mutation and buthionine sulphoximine, an inhibitor of γ-glutamylcysteine synthetase, enhances first peak of the ozone-induced biphasic [Ca^2+^]_cyt_ response (Evans et al., 2005). These results suggest that GSH pools control sensitivity of plasma membrane Ca^2+^ permeable channels to ROS and downstream Ca^2+^ signals in plant cells.

**Figure 6:**
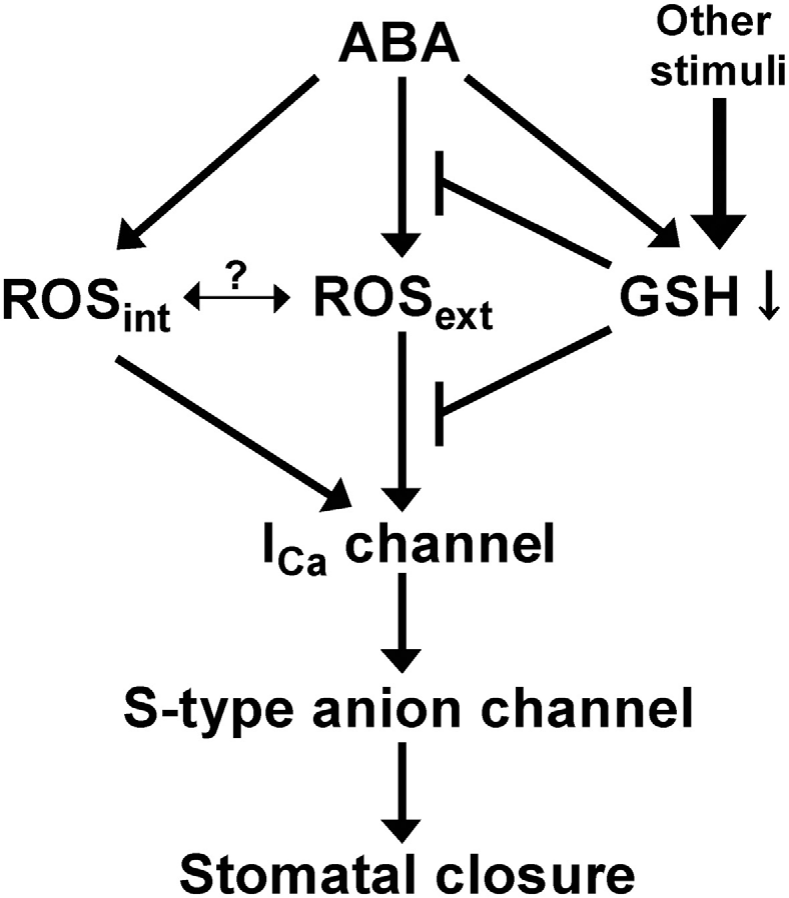
**A simplified model of GSH regulation of guard cell ABA signaling**.

Consistent with the enhancement of I_Ca_ channel response to ROS, the *cad2-1* mutant exhibits enhanced H_2_O_2_-induced stomatal closure (Figure 4). Note that depletion of GSH in guard cell cytosol is induced by ABA (Okuma et al., 2011), but not by H_2_O_2_ (Akter et al., 2013). These results imply that ABA sensitizes guard cells to ROS by decreasing GSH content via a pathway distinct from the ABA-mediated ROS production pathway (Figure 6).

Previously we reported identical cytosolic ROS accumulation induced by ABA in cytosol of wild-type and *cad2-1* guard cells using 2′,7′-dichlorodihydrofluorescein diacetate (Okuma et al., 2011). It has been suggested that apoplastic ROS signals are also involved in regulation of stomatal movement (An et al., 2008; Khokon et al., 2011; Hossain et al., 2013). In this study we monitored ROS accumulation in whole leaves using DAB. ABA induced ROS accumulation in whole leaves of the *cad2-1* mutant but not in leaves of wild type (Figure 5). These results suggest that GSH depletion by the *cad2-1* mutation affects ROS homeostasis in apoplastic space rather than that in guard cells during ABA-induced stomatal closure.

Based on the findings obtained in this study, we present one simplified signal model shown in Figure 6. ABA decreases guard cell GSH content via ROS-independent pathway (Akter et al., 2013). The decreased GSH content causes significant ROS accumulation in apoplast (Figure 5) and also sensitizes guard cell I_Ca_ channels to apoplastic ROS (Figure 3) by unknown mechanism, resulting in enhanced stomatal response to ABA. In wild-type leaves, ABA decreases GSH content (Okuma et al., 2011) but does not induce significant apoplastic ROS accumulation (Figure 5), suggesting that the apoplastic ROS signal is employed to modulate ABA responsiveness of guard cells by other stimuli rather than by ABA signaling itself. Molecular mechanisms of how GSH regulates ROS sensitivity to plasma membrane Ca^2+^ permeable channels and apoplastic ROS homeostasis during ABA-induced stomatal closure would be investigated in the future.

### Conflict of interest statement

The authors declare that the research was conducted in the absence of any commercial or financial relationships that could be construed as a potential conflict of interest.

## Supplementary material

Figure S1 **GSHmee attenuates enhanced stomatal response to H_2_O_2_ of the *cad2-1*mutant.** Detached rosette leaves of the *cad2-1* mutant were incubated on stomatal assay buffer (see Materials and Methods) with or without 10 μM GSHmee for 2 h in the light, followed by the addition of 100 μM H_2_O_2_. Stomatal apertures were measured 2 h after H_2_O_2_ application. Twenty averages from three independent experiments (60 total stomata per bar) are shown. The bars represent the mean ± *SE* values.

## References

[B1] Akter N. Okuma E. Sobahan M. A. Uraji M. Munemasa S. Nakamura Y. (2013). Negative regulation of methyl jasmonate-induced stomatal closure by glutathione in *Arabidopsis*. J. Plant Growth Regul. 32, 208–215 10.1007/s00344-012-9291-7

[B2] Akter N. Sobahan M. A. Hossain M. A. Uraji M. Nakamura Y. Mori I. C. (2010). The involvement of intracellular glutathione in methyl jasmonate signaling in *Arabidopsis* guard cells. Biosci. Biotechnol. Biochem. 74, 2504–2506 10.1271/bbb.100513 21150111

[B3] Akter N. Sobahan M. A. Uraji M. Ye W. Hossain M. A. Mori I. C. (2012). Effects of depletion of glutathione on abscisic acid- and methyl jasmonate-induced stomatal closure in *Arabidopsis thaliana*. Biosci. Biotechnol. Biochem. 76, 2032–2037 10.1271/bbb.120384 23132563

[B4] Allan A. C. Fricker M. D. Ward J. L. Beale M. H. Trewavas A. J. (1994). Two transduction pathways mediate rapid effects of abscisic acid in *Commelina* guard cells. Plant Cell 6, 1319–1328 10.2307/3869829 12244274 PMC160523

[B5] Allen G. J. Kuchitsu K. Chu S. P. Murata Y. Schroeder J. I. (1999). *Arabidopsis abi1-1* and *abi2-1* phosphatase mutations reduce abscisic acid-induced cytosolic calcium rises in guard cells. Plant Cell 11, 1785–1798 10488243 10.1105/tpc.11.9.1785 PMC144302

[B6] An Z. Jing W. Liu Y. L. Zhang W. H. (2008). Hydrogen peroxide generated by copper amine oxidase is involved in abscisic acid-induced stomatal closure in *Vicia faba*. J. Exp. Bot. 59, 815–825 10.1093/jxb/erm370 18272918

[B7] Bindschedler L. V. Dewdney J. Blee K. A. Stone J. M. Asai T. Plotnikov J. (2006). Peroxidase-dependent apoplastic oxidative burst in *Arabidopsis* required for pathogen resistance. Plant, J. 47, 851–863 10.1111/j.1365-313X.2006.02837.x 16889645 PMC3233234

[B8] Brandt B. Brodsky D. E. Xue S. Negi J. Iba K. Kangasjärvi J. (2012). Reconstitution of abscisic acid activation of SLAC1 anion channel by CPK6 and OST1 kinases and branched ABI1 PP2C phosphatase action. Proc. Natl. Acad. Sci. U.S.A. 109, 10593–10598 10.1073/pnas.1116590109 22689970 PMC3387046

[B9] Evans N. H. McAinsh M. R. Hetherington A. M. Knight M. R. (2005). ROS perception in *Arabidopsis thaliana*: the ozone-induced calcium response. Plant J. 41, 615–626 10.1111/j.1365-313X.2004.02325.x 15686524

[B10] Fujii H. Chinnusamy V. Rodrigues A. Rubio S. Antoni R. Park S. Y. (2009). *In vitro* reconstitution of an abscisic acid signalling pathway. Nature 462, 660–664 10.1038/nature08599 19924127 PMC2803041

[B11] Geiger D. Scherzer S. Mumm P. Marten I. Ache P. Matschi S. (2010). Guard cell anion channel SLAC1 is regulated by CDPK protein kinases with distinct Ca^2+^ affinities. Proc. Natl. Acad. Sci. U.S.A. 107, 8023–8028 10.1073/pnas.0912030107 20385816 PMC2867891

[B12] Geiger D. Scherzer S. Mumm P. Stange A. Marten I. Bauer H. (2009). Activity of guard cell anion channel SLAC1 is controlled by drought-stress signaling kinase-phosphatase pair. Proc. Natl. Acad. Sci. U.S.A. 106, 21425–21430 10.1073/pnas.0912021106 19955405 PMC2795561

[B13] Gilroy S. Fricker M. D. Read N. D. Trewavas A. J. (1991). Role of calcium in signal transduction of Commelina guard cells. Plant Cell 3, 333–344 10.2307/3869209 12324599 PMC160004

[B14] Grabov A. Blatt M. R. (1998). Membrane voltage initiates Ca^2+^ waves and potentiates Ca^2+^ increases with abscisic acid in stomatal guard cells. Proc. Natl. Acad. Sci. U.S.A. 95, 4778–4783 10.1073/pnas.95.8.4778 9539815 PMC22567

[B15] Hamilton D. W. A. Hills A. Köhler B. Blatt M. R. (2000). Ca^2+^ channels at the plasma membrane of stomatal guard cells are activated by hyperpolarization and abscisic acid. Proc. Natl. Acad. Sci. U.S.A. 97, 4967–4972 10.1073/pnas.080068897 10781106 PMC18341

[B16] Hossain M. S. Ye W. Hossan M. A. Okuma E. Uraji M. Nakamura Y. (2013). Glucosinolate degradation products, isothiocyanates, nitriles, and thiocyanates, induce stomatal closure accompanied by peroxidase-mediated reactive oxygen species production in *Arabidopsis thaliana*. Biosci. Biotechnol. Biochem. 77, 977–983 10.1271/bbb.120928 23649257

[B17] Hu H. Boisson-Dernier A. Israelsson-Nordström M. Böhmer M. Xue S. Ries A. (2010). Carbonic anhydrases are upstream regulators of CO2-controlled stomatal movements in guard cells. Nat. Cell Biol. 12, 87–93 10.1038/ncb2009 20010812 PMC2906259

[B18] Jahan M. S. Ogawa K. Nakamura Y. Shimoishi Y. Mori I. C. Murata Y. (2008). Deficient glutathione in guard cells facilitates abscisic acid-induced stomatal closure but does not affect light-induced stomatal opening. Biosci. Biotechnol. Biochem. 72, 2795–2798 10.1271/bbb.80407 18838781

[B19] Khokon A. R. Okuma E. Hossain M. A. Munemasa S. Uraji M. Nakamura Y. (2011). Involvement of extracellular oxidative burst in salicylic acid-induced stomatal closure in *Arabidopsis*. Plant Cell Environ. 34, 434–443 10.1111/j.1365-3040.2010.02253.x 21062318

[B20] Kwak J. M. Mori I. C. Pei Z. M. Leonhardt N. Torres M. A. Dangl J. L. (2003). NADPH oxidase *AtrbohD* and *AtrbohF* genes function in ROS-dependent ABA signaling in *Arabidopsis*. EMBO J. 22, 2623–2633 10.1093/emboj/cdg277 12773379 PMC156772

[B21] Lee S. C. Lan W. Buchanan B. B. Luan S. (2009). A protein kinase-phosphatase pair interacts with an ion channel to regulate ABA signaling in plant guard cells. Proc. Natl. Acad. Sci. U.S.A. 106, 21419–21424 10.1073/pnas.0910601106 19955427 PMC2795491

[B22] Linder B. Raschke K. (1992). A slow anion channel in guard cells, activating at large hyperpolarization, may be principal for stomatal closing. FEBS Lett. 313, 27–30 10.1016/0014-5793(92)81176-M 1385219

[B23] Ma Y. Szostkiewicz I. Korte A. Moes D. Yang Y. Christmann A. (2009). Regulators of PP2C phosphatase activity function as abscisic acid sensors. Science 324, 1064–1068 10.1126/science.1172408 19407143

[B24] Marten H. Konrad K. R. Dietrich P. Roelfsema M. R. G. Hedrich R. (2007). Ca^2+^-dependent and -independent abscisic acid activation of plasma membrane anion channels in guard cells of *Nicotiana tabacum*. Plant Physiol. 143, 28–37 10.1104/pp.106.092643 17142476 PMC1761993

[B25] Maruta T. Tanouchi A. Tamoi M. Yabuta Y. Yoshimura K. Ishikawa T. (2010). Arabidopsis chloroplastic ascorbate peroxidase isoenzymes play a dual role in photoprotection and gene regulation under photooxidative stress. Plant Cell Physiol. 51, 190–200 10.1093/pcp/pcp177 20007290

[B26] May M. J. Vernoux T. Leaver C. van Montagu M. Inze D. (1998). Glutathione homeostasis in plants: implications for environmental sensing and plant development. J. Exp. Bot. 49, 649–667 10.1093/jexbot/49.321.649

[B27] McAinsh M. R. Brownlee C. Hetherington A. M. (1990). Abscisic acid-induced elevation of guard-cell cytosolic Ca^2+^ precedes stomatal closure. Nature 343, 186–188 10.1038/343186a011607281

[B28] Miao Y. Lv D. Wang P. Wang X. C. Chen J. Miao C. (2006). Arabidopsis glutathione peroxidase functions as both a redox transducer and a scavenger in abscisic acid and drought stress responses. Plant Cell 18, 2749–2766 10.1105/tpc.106.044230 16998070 PMC1626619

[B29] Munemasa S. Hossain M. A. Nakamura Y. Mori I. C. Murata Y. (2011). The Arabidopsis calcium-dependent protein kinase, CPK6, functions as a positive regulator of methyl jasmonate signaling in guard cells. Plant Physiol. 155, 553–561 10.1104/pp.110.162750 20978156 PMC3075756

[B30] Munemasa S. Oda K. Watanabe-Sugimoto M. Nakamura Y. Shimoishi Y. Murata Y. (2007). The *coronatine-insensitive 1* mutation reveals the hormonal signaling interaction between abscisic acid and methyl jasmonate in *Arabidopsis* guard cells. Specific impairment of ion channel activation and second messenger production. Plant Physiol. 143, 1398–1407 10.1104/pp.106.091298 17220365 PMC1820907

[B31] Murata Y. Pei Z. M. Mori I. C. Schroeder J. I. (2001). Abscisic acid activation of plasma membrane Ca^2+^ channels in guard cells requires cytosolic NAD(P)H and is differentially disrupted upstream and downstream of reactive oxygen species production in *abi1-1* and *abi2-1* protein phosphatase 2C mutants. Plant Cell 13, 2513–2523 10.1105/tpc.13.11.2513 11701885 PMC139468

[B32] Negi J. Matsuda O. Nagasawa T. Oba Y. Takahashi H. Kawai-Yamada M. (2008). CO_2_ regulator SLAC1 and its homologues are essential for anion homeostasis in plant cells. Nature 452, 483–486 10.1038/nature06720 18305482

[B33] Noctor G. Foyer C. H. (1998). Ascorbate and glutathione: keeping active oxygen under control. Annu. Rev. Plant Physiol. Plant Mol. Biol. 49, 249–279 10.1146/annurev.arplant.49.1.249 15012235

[B34] Okuma E. Jahan M. S. Munemasa S. Hossain M. A. Muroyama D. Islam M. M. (2011). Negative regulation of abscisic acid-induced stomatal closure by glutathione in *Arabidopsis*. J. Plant Physiol. 168, 2048–2055 10.1016/j.jplph.2011.06.002 21764168

[B35] Park S. Y. Fung P. Nishimura N. Jensen D. R. Fujii H. Zhao Y. (2009). Abscisic acid inhibits type 2C protein phosphatases via the PYR/PYL family of START proteins. Science 324, 1068–1071 10.1126/science.1173041 19407142 PMC2827199

[B36] Pei Z. M. Kuchitsu K. Ward J. M. Schwarz M. Schroeder J. I. (1997). Differential abscisic acid regulation of guard cell slow anion channels in Arabidopsis wild-type and *abi1* and *abi2* mutants. Plant Cell 9, 409–423 10.2307/3870491 9090884 PMC156927

[B37] Pei Z. M. Murata Y. Benning G. Thomine S. Klüsener B. Allen G. J. (2000). Calcium channels activated by hydrogen peroxide mediate abscisic acid signalling in guard cells. Nature 406, 731–734 10.1038/35021067 10963598

[B38] Schroeder J. I. Hagiwara S. (1989). Cytosolic calcium regulates ion channels in the plasma membrane of Vicia faba guard cells. Nature 338, 427–430 10.1038/338427a0

[B39] Schroeder J. I. Hagiwara S. (1990). Repetitive increases in cytosolic Ca^2+^ of guard cells by abscisic acid activation of nonselective Ca^2+^ permeable channels. Proc. Natl. Acad. Sci. U.S.A. 87, 9305–9309 10.1073/pnas.87.23.9305 2174559 PMC55153

[B40] Schroeder J. I. Keller B. U. (1992). Two types of anion channel currents in guard cells with distinct voltage regulation. Proc. Natl. Acad. Sci. U.S.A. 89, 5025–5029 10.1073/pnas.89.11.5025 1375754 PMC49221

[B41] Siegel R. S. Xue S. Murata Y. Yang Y. Nishimura N. Wang A. (2009). Calcium elevation-dependent and attenuated resting calcium-dependent abscisic acid induction of stomatal closure and abscisic acid-induced enhancement of calcium sensitivities of S-type anion and inward-rectifying K channels in *Arabidopsis* guard cells. Plant J. 59, 207–220 10.1111/j.1365-313X.2009.03872.x 19302418 PMC2827207

[B42] Vahisalu T. Kollist H. Wang Y. F. Nishimura N. Chan W. Y. Valerio G. (2008). SLAC1 is required for plant guard cell S-type anion channel function in stomatal signalling. Nature 452, 487–491 10.1038/nature06608 18305484 PMC2858982

