# Supplementary figures and images for: Regulation of reactive oxygen species-mediated abscisic acid signaling in guard cells and drought tolerance by glutathione
Source: Front Plant Sci. 2013 Nov 20;4:472. doi: 10.3389/fpls.2013.00472 (PMC3834289; doi:10.3389/fpls.2013.00472)

Figure A1

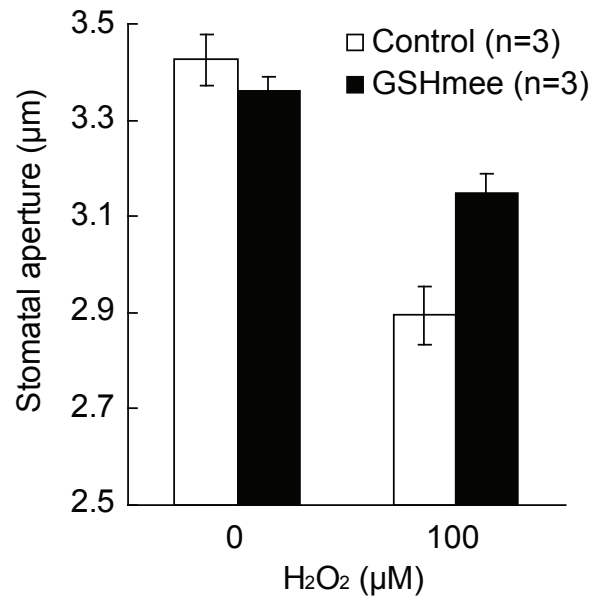

Supplement: Figure S1 — GSHmee attenuates enhanced stomatal response to H2O2 of the cad2-1mutant. Detached rosette leaves of the cad2-1 mutant were incubated on stomatal assay buffer (see Materials and Methods) with or without 10 μM GSHmee for 2 h in the light, followed by the addition of 100 μM H2O2. Stomatal apertures were measured 2 h after H2O2 application. Twenty averages from three independent experiments (60 total stomata per bar) are shown. The bars represent the mean ± SE values. [file Presentation1.PDF]
